# The Effect of Adhesive Layer Thickness on Joint Static Strength

**DOI:** 10.3390/ma14061499

**Published:** 2021-03-18

**Authors:** Marek Rośkowicz, Jan Godzimirski, Andrzej Komorek, Michał Jasztal

**Affiliations:** 1 Faculty of Mechatronics, Armament and Aerospace, Military University of Technology, 00-908 Warsaw, Poland; marek.roskowicz@wat.edu.pl (M.R.); jan.godzimirski@wat.edu.pl (J.G.); michal.jasztal@wat.edu.pl (M.J.); 2 Faculty of Aviation, Polish Air Force University, 08-521 Dęblin, Poland

**Keywords:** adhesive joint, adhesive bond strength, adhesive layer thickness

## Abstract

One of the most relevant geometrical factors defining an adhesive joint is the thickness of the adhesive layer. The influence of the adhesive layer thickness on the joint strength has not been precisely understood so far. This article presents simplified analytical formulas for adhesive joint strength and adhesive joint coefficient for different joint loading, assuming, inter alia: linear-elastic strain of adhesive layer, elastic strain of adherends and only one kind of stress in adhesive. On the basis of the presented adhesive joint coefficient, the butt joint was selected for the tests of the influence of adhesive thickness on the adhesive failure stress. The tests showed clearly that with an increase in the thickness of the tested adhesive layers (up to about 0.17 mm), the value of their failure stress decreased quasi linearly. Furthermore, some adhesive joints (inter alia subjected to shearing) may display the optimum value of the thickness of the adhesive layer in terms of the strength of the joint. Thus, the aim of this work was to explain the phenomenon of optimal adhesive layer thickness in some types of adhesive joints. The verifying test was conducted with use of single simple lap joints. Finally, with the use of the FE method, the authors were able to obtain stresses in the adhesive layers of lap joints for loads that destroyed that joints in the experiment, and the FEM-calculated failure stresses for lap joints were compared with the adhesive failure stresses determined experimentally using the butt specimens. Numerical calculations were conducted with the use of the continuum mechanics approach (stress-based), and the non-linear behavior of the adhesive and plastic strain of the adherends was taken into account.

## 1. Introduction

Adhesive bonds are frequently used in numerous industrial sectors [1,2]. Adhesive bonding is a particularly attractive assembly technique for application in which weight gain is at a premium, e.g., air transport [3]. In the aeronautical industry, adhesive bonds are most frequently applied in the manufacturing of sandwich constructions, fiber metal laminates and fatigue-resistant hybrid adhesive-riveted joints. In pure adhesive joints, a wide zone can be used to transmit the load, and no holes are used; thus, a reduction in weight can be obtained. However, the reliability of adhesively bonded joints needs to be properly addressed during their design. The strength of adhesive joints depends upon many constructional and technological factors and service conditions. The geometry of the adhesive joint, the sort of joint materials used (both adhesive and adherends), and the load manner are ranked as constructional factors. The geometry of the structural adhesive joints is a very important aspect in the design of the joint. One of the most relevant geometrical factors is the thickness of the adhesive layer.

Numerous investigations have been carried out in order to determine the influence of adhesive thickness on the strength of adhesive joints [4,5,6,7,8,9,10,11,12,13,14]. However, the effect of the adhesive thickness on the bond strength, even in single lap joints, is still not precisely understood.

As early as sixty years ago, Alner [4] and Winter [5] indicated that the dependence of adhesive joint strength on adhesive thickness was different from the results shown by analytical formulas. According to the analytical formulas, an increase in adhesive thickness should lead to an increase in the strength of adhesive joints subjected to shearing. In fact, usually, the strength of such joints decreases with increasing adhesive thickness if it is more than the so-called optimum thickness. For most adhesives, the optimal adhesive thickness is between 0.05 and 0.15 mm [1].

Grant et al. [6] verified that lap joints under tension are very sensitive to adhesive thickness. They explained that as the bond line thickness increases, there is an increase in the bending stress, since the bending moment increases. Consequently, the strength of the joint is reduced. Additionally, da Silva et al. [7] verified that lap shear strength decreases as the adhesive thickness increases from 0.5 to 2 mm.

Naito et al. [8] researched the effect of adhesive thickness on the tensile and shear strength of polyimide adhesive. Their experimental test results indicated that the tensile strength of butt joints decreases with increasing adhesive thickness, and the shear strength of single lap joints is almost constant, regardless of adhesive thickness (0.1–1 mm), although a lot of scatter was observed in the tensile and shear strength of the tested adhesive.

Xu and Wei [9] researched the influence of adhesive thickness on the cohesive properties and overall strength of metallic adhesive bonding structures, employing the cohesive zone model to equivalently simulate adhesive layers with various thicknesses. The obtained results showed that both the cohesive parameters and the overall strength of metallic adhesive were highly dependent on the adhesive thickness, and the variations in overall strength resulting from the various thickness exhibited discrepancies due to the toughness and strain hardening capacity of the adhesive.

Bezemer et al. [10] tested the impact strength of the adhesive. They proved that there is an optimum adhesive thickness at different test speeds. The optimum thickness depends both on the adhesive used and on the test speed. Only for tough adhesives did increased adhesive thickness give a better impact behavior.

Arends et al. [11] took up the attempt to determine optimum adhesive thickness for structural adhesive joints. They performed experimental tests and carried out analyses concerning the influence of adhesive thickness on the tensile lap-shear strength of single overlap joints. For adhesive thicknesses between 0.4 and 0.8 mm, it was found that the failure mode was essentially cohesive, and the value of the strength increased as the adhesive thickness was reduced. For adhesive thicknesses less than 0.4 mm, it was noted that the failure mode was essentially adhesive, and the shear strength showed higher values, but with typical deviation. Because the cohesive failure mode guaranteed the greater reliability of the adhesive joint under the foreseeable working conditions, the optimal adhesive thickness for the tested adhesive should be maintained between 0.4 and 0.5 mm.

Marzi et al. [12] researched the influence of adhesive thickness on the fracture energy of the crash-optimized high-strength adhesive. All of the methods used by them gave virtually the same fracture energy for small layer thicknesses. Here, the fracture energy increased with increasing layer thickness. For larger thicknesses, the results obtained from different methods deviated.

Praksah et al. [13] investigated the influence of adhesive thickness on the high-velocity impact performance of composite targets. The research proved that parameters such as target deformation, energy transformation, depth of penetration and profile deflection of the back plate were dependent to a high degree on the adhesive layer.

Cognard et al. [14] experimentally tested and numerically analyzed the influence of relatively thick adhesive thicknesses (until 1.3 mm) on the strength and edge effects of TAST specimens. They showed that an increase in the adhesive thickness increases the influence of edge effect and increases the risk of crack initiation near the free edges of adhesive. This conclusion seems to be applicable to joints loaded in a similar fashion to the TAST specimens.

In addition to the experimental approach, prediction of the strength of adhesively bonded joints can be performed with use of three main numerical approaches: the continuum mechanics approach (stress-based), the fracture mechanics approach, and the damage mechanics approach [15].

In the continuum mechanics approach, the adhesive and adherends are modeled using continuum elements, assuming that the adhesive is perfectly bonded to the adherends. The assumption of a perfect bond means that the finite element analysis takes no account of the adhesion properties of the interface. This type of modeling approach makes it possible to take into account the influence of the thickness of the adhesive layer on adhesively bonded joint strength [16].

The fracture mechanics approach uses an energy parameter (toughness) as the failure criterion. Prediction of the joint strength is based on linear elastic fracture mechanics, which relies on the existence of a crack and linear elasticity [17,18]. However, well-manufactured joints may not have macroscopic cracks. Furthermore, large-scale plasticity in the adherends limits the use of the linear elastic fracture mechanics approach in numerical calculations.

The progressive damage approach can be implemented using either local or continuum approaches. In the continuum approach, the damage is modeled over a finite region. The local approach, in which the damage is confined to a zero thickness of the surface, is often referred to as the cohesive zone approach (CZM). The CZM approach is used to simulate the macroscopic damage along predefined crack path by specification of a traction–separation response between initially coincident nodes on either side of the predefined crack path [19]. In the FEM strength calculations of adhesively bonded joints with the use of “cohesive” elements (CZM), the problem becomes to assign appropriate values to the parameters of such elements. The analyses of the authors of the article [20] show that this requires experimental tests and numerical calculations for a specific adhesive, the method of preparing the surface for adhesive and specific thickness of the joint. “Cohesive” elements (CZM) have no thickness and make it possible to determine only normal stresses perpendicular to the bonded surfaces and shear stresses (normal stresses occurring in the adhesive plane are ignored). For this reason, they have been found to be unsuitable for the numerical analysis of the influence of the adhesive layer thickness on static strength.

The purpose of the numerical calculations in this paper is to obtain stresses in the adhesive layers of lap joints for loads that destroy that joints in the experiment, and compare the FEM-calculated failure stresses of the lap joints with the adhesive failure stresses determined experimentally. Therefore, the authors decided to apply the continuum mechanics approach.

The general topic of the present paper is the analysis of the influence of the thickness of the adhesive layer on the strength of differently loaded adhesive joints. However, the main aim of the work is to explain the phenomenon of optimal adhesive layer thickness in some types of adhesive joints. The article presents simplified analytical formulas for adhesive joint strength for different joint loading and elastic properties of adhesive and adherends, calculated on the basis of Refs. [21,22,23,24,25,26]. The experimental tests were conducted with the use of the butt joints and single simple lap joints. Finally, FE method calculations were carried out within the range of plastic deformations of both adhesive and adherend in order to verify the adhesive failure stress dependence on adhesive thickness.

## 2. Analytical Calculations of the Strength of Adhesive Joint

In the analytical calculations, the assumption may be made that the strength of the adhesive joint is given by the formula:

(1) $$P=\tau_{n}\cdot A\cdot \;\psi$$

where:

*τ_n_*—breaking stresses of the adhesive layer dependent on the sort of adhesive, the sort of adherend material, the adhesive bonding technology (among others surface preparation), and the adhesive thickness;

*A*—area of the adhesive layer;

*ψ*—joint coefficient, dependent on the joint geometry, load manner, mechanical properties of adhesive and mechanical properties of the adherends.

The joint coefficient may be determined analytically. Even for numerous simplifications, including

- two-dimensional models,
- elastic strain of the adherends,
- linear-elastic strain of the adhesive layer,
- constant stress along thickness of the adhesive layer, and
- only one stress component in the adhesive,

the joint coefficient for lap joints subjected to shearing is given by a quite complicated formula resulting from Volkersen’s theory:

(2) $$\psi =\sqrt{\frac{W}{\Delta }}\frac{\sinh\sqrt{\Delta W}}{\cosh\sqrt{\Delta W}+W-1}$$

where:

$\Delta =\frac{G_{k}l^{2}}{\delta_{k}E_{2}\delta_{2}}$, $W=\frac{E_{1}\delta_{1}+E_{2}\delta_{2}}{E_{1}\delta_{1}}$

where:

*E*—Youngs’ modulus of the adherends,

*G_k_*—shear modulus of the adhesive (adhesive layer),

*l*—length of the adhesive layer,

*δ*—thickness of the adherends,

*δ_k_*—thickness of the adhesive layer.

Considering the geometry of most of the lap joints used in engineering, it is possible to simplify Equation (1) to:

(3) $$\Psi =\sqrt{\frac{W}{A}}=\sqrt{\frac{E_{1}\delta_{1}+E_{2}\delta_{2}}{E_{1}\delta_{1}}}\times \sqrt{\frac{\delta_{k}E_{2}\delta_{2}}{G_{k}l^{2}}}$$

Assuming that both adherends have equal longitudinal rigidity, it is possible to make additional simplifications. In Table 1, simplified formulas of joint coefficients (calculated on the basis of papers [21,22,23,24,25,26]) are presented for different models of adhesive joint loading.

In Table 1, only failure shear stresses are presented. It follows from this that the maximal principal positive stresses hypothesis is useful to evaluate the effort in the adhesive layer with acceptable accuracy, and for this hypothesis, the failure shear stresses are equal to the failure normal stresses (*σ_n_* = *τ_n_*) [27]. This results from the secular equation:

(4) $$\begin{array}{cc} \sigma^{3}-\sigma^{2}\left(\sigma_{x}+\sigma_{y}+\sigma_{z}\right)+\sigma \left(\sigma_{x}\sigma_{y}+\sigma_{y}\sigma_{z}+\sigma_{z}\sigma_{x}-\tau_{xy}^{2}-\tau_{yz}^{2}-\tau_{zx}^{2}\right) & \\ -\left(\sigma_{x}\sigma_{y}\sigma_{z}-\sigma_{x}\tau_{yz}^{2}-\sigma_{y}\tau_{zx}^{2}-\sigma_{z}\tau_{xy}^{2}+2\tau_{xy}\tau_{yz}\tau_{zx}\right) & =0 \end{array}$$

where *σ*—principal stress, *σ_x_*, *σ_y_*, *σ_z_*—normal stress, *τ_xy_*, *τ_xz_*, *τ_yz_*—shear stress

If *σ_x_* = 0, *σ_y_* = 0, *σ_z_* = 0, *τ_yz_* = 0 and *τ_zx_* = 0, then

(5) $$\sigma_{I}=\tau_{xy}$$

where *P*—force, *M_g_* —bending moment, *M_s_*—twisting moment, *d*—diameter, *e*—eccentricity, *E*—Youngs’ modulus of the adherends, *E_k_*—Young’ modulus of the adhesive, *G_k_*—shear modulus of the adhesive (adhesive layer), *l*—length of the adhesive layer, *δ*—thickness of the adherends, *δ_k_*—thickness of the adhesive layer, *A*—area of the adhesive layer.

On the basis of the formulas shown in Table 1, it can be seen that only the strength of joints subjected to pure shearing (No. 4), to tension (No. 11) and to cleaving (No. 12,13) are not dependent upon the adhesive layer thickness. The strength of the other joints (the most important for engineering subjected to shearing) is dependent upon the adhesive thickness, and this dependence can be described by the following formula:

(6) $$P,M_{s},M_{g}=C_{i}\sqrt{\delta_{k}}$$

where $C_{i}$—constant value for specific type of joint.

**Figure 1:**
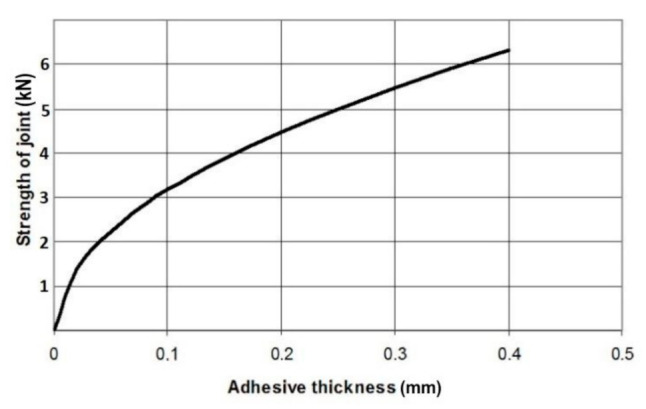
Theoretical dependence of lap adhesive joint strength upon adhesive layer thickness when failure stress of the adhesive layer is not dependent on adhesive layer thickness (vertical axis non-dimensional).

According to Equation (6), the strength of the majority of adhesive joints should increase along with an increase in adhesive thickness, as shown in Figure 1.

The experimental tests do not verify this dependence on account of the fact that the failure stress of the adhesive is dependent upon the adhesive thickness. Adams and Peppiatt [28] attribute the decrease in joint strength with adhesive thickness to the fact that thicker bond lines contain more defects, such as voids and microcracks. This theory seems to be very probable, although there are many other theories.

## 3. Experimental Tests

**Figure 2:**
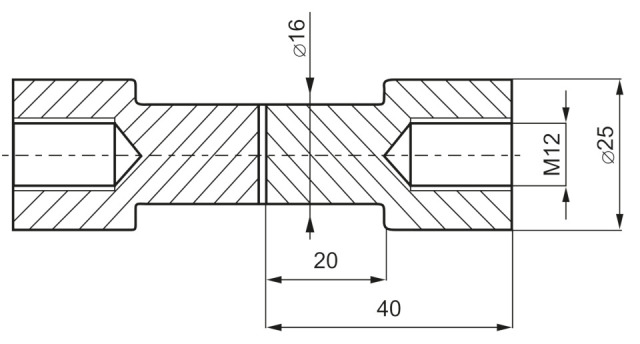
The specimen used in the test for estimating the dependence of adhesive failure stress upon adhesive thickness.

To determine the influence of adhesive thickness upon the failure stress of the adhesive layer, an experiment was carried out with the butt specimens, as shown in Figure 2. The simple butt joint was selected for the tests, because its joint coefficient is not dependent upon adhesive thickness (see Table 1).

**Figure 3:**
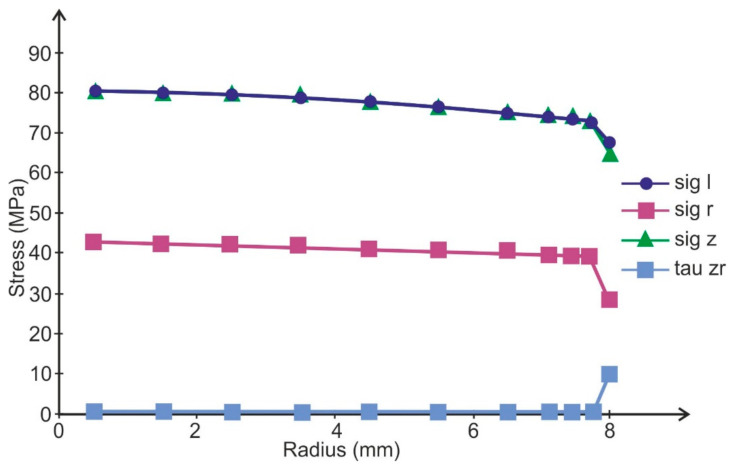
Stresses distribution in the adhesive layer of the butt specimen (sig I—maximal principal stress, sig r—radial stress equal circumferential stress, sig z—axial stress, tau zr—shear stress, the adhesive was modeled by three layers of elements).

The FEM calculations conducted showed that in the adhesive layer of such specimens, the distribution of stresses is approximately uniform (Figure 3).

The bonded surfaces of all specimens were prepared by pickling in aqueous solution of sulfuric acid and chromic acid at 60 °C. In the strength tests, six specimens were prepared for every measuring point. The results were elaborated statistically: Student-Fisher’s method was applied to calculate the confidence interval for the level α = 0.95. To obtain the required adhesive thickness, two thin wires of suitable thickness were placed in uncured adhesive layers. The thinnest adhesive thicknesses (about 0.02 mm) were obtained without distance wires. The adhesive layers were cured at a pressure of about 0.8 MPa. The adhesive thicknesses were estimated by measuring similarly adhesively bonded single simple lap specimens which thickness were measured by micrometer. The thickest bond line was 0.3 mm.

The tests were conducted for Epidian 57 adhesive (CIECH Resins, Nowa Sarzyna, Poland) cured with Z-1 hardener (CIECH Resins, Nowa Sarzyna, Poland) for 1 h at 60 °C and Araldite AW126H (Huntsman Advanced Materials, Duxford, UK) cured with HY994 hardener (Huntsman Advanced Materials, Duxford, UK) for 1 h at 80 °C. The failure stresses were calculated using the following formula:

(7) $$\tau_{n}=\sigma_{n}=\frac{P}{A}$$

where *P*—average breaking force, *A*—area of adhesive layer.

**Figure 4:**
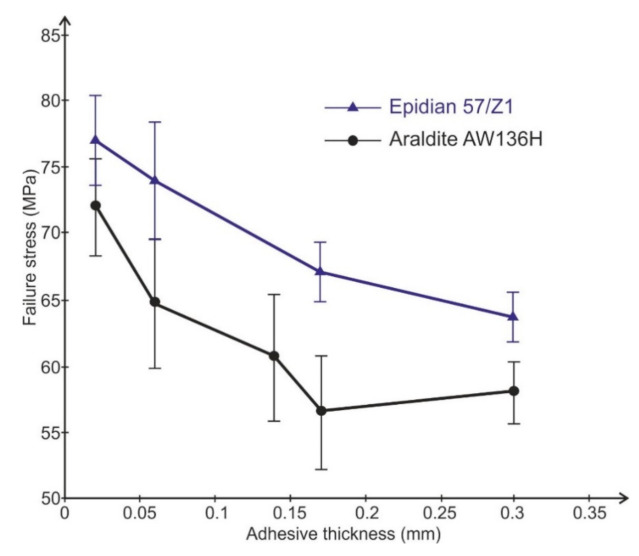
Dependence of adhesive failure stress upon adhesive thickness determined for two adhesives.

The results of the tests are presented in Figure 4.

The tests showed explicitly that value of adhesive failure stress are dependent upon the adhesive thickness. Along with the increase in the adhesive thicknesses to about 0.17 mm, the value of failure stress decreases in a quasi-linear fashion for the tested adhesive. A similar dependence of failure shear stress upon adhesive thickness was obtained for heading joints with two sleeves subjected to shearing [29] and normal stress for tensile butt joints [8].

The linear dependence of adhesive failure stresses upon adhesive thickness can be described by the following formula:

(8) $$\sigma_{n}=\tau_{n}=B-D\cdot \delta_{k}$$

where *B* and *D*—constants for each adhesive dependent on surface preparation manner for adhesive bending.

The insertion of Equation (8), e.g., into the formula for loading manner 1, shown in Table 1, makes it possible to obtain the dependence of the strength of the joint subjected to shearing on the adhesive layer thickness:

(9) $$P=(B-D\cdot \delta_{k})A\sqrt{\frac{2E\delta \delta_{k}}{l^{2}G_{K}}}$$

Function *P* = *P*(*δ_k_*) has an extreme (maximum) for:

(10) $$\delta_{k}=\frac{B}{3D}$$

and this formula describes the optimum thickness value of the adhesive layer for load manner 1, shown in Table 1. The same formula describes the optimum thickness value of the adhesive layer for manners of adhesive bond loading 2, 3, 5, 7, 9 and 14, shown in Table 1.

For adhesive bonds loaded according to manner 6 (shown in Table 1), the strength as a function of adhesive thickness is described by the following formula:

(11) $$P=(B-D\cdot \delta_{k})A\sqrt[{4}]{\frac{E\delta^{3}\delta_{k}}{96l^{4}E_{k}}}$$

Equation (11) has a maximum for:

(12) $$\delta_{k}=\frac{3B}{7D}$$

The same formula describes the optimum thickness value of the adhesive layer for loading manners 8 and 10, shown in Table 1.

Due to the above, it seems that some adhesive joints may have an optimum adhesive thickness. The optimum thickness value of the adhesive layer shouldn’t be dependent upon the thickness of the adherends, the Young’s modulus of the bonded materials, or the Young’s modulus of the adhesive, but it should be dependent upon loading manner.

On the basis of Figure 3, the linear dependences of failure stress on adhesive thickness (*τ_n_* = *τ*(*δ_k_*)) were calculated as follows for the Epidian 57 adhesive:

(13) $$\tau_{n}=76.9-160.1\;\;\;\delta_{k}\cdot \mathrm{MPa}$$

and for Araldite AV 136H:

(14) $$\tau_{n}=72.2-240.8\;\;\delta_{k}\cdot \mathrm{MPa}$$

By inserting Dependencies (13) and (14) into Equation (10), we can calculate the values of the optimal thickness of the adhesive layer for the two tested adhesives. Therefore, the optimal adhesive thickness for joints subjected to shearing bonded with Epidian 57 adhesive should be equal, at about:

(15) $$\delta_{kopt}=\frac{B}{3D}=\frac{76.9}{3\cdot 160.1}\approx 0.16\;\;mm$$

and, in turn, for joints subjected to shearing bonded with Araldite AV 136 H adhesive:

(16) $$\delta_{kopt}=\frac{72.2}{3\cdot 240.8}\approx 0.1\;\;mm$$

**Figure 5:**
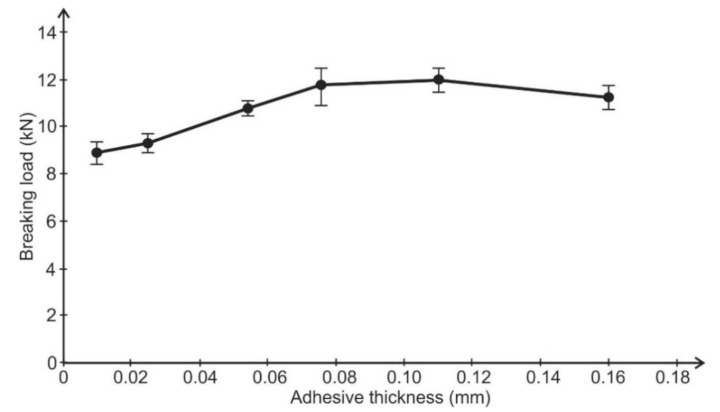
Dependence of lap joint strength on adhesive thickness (Epidian 57/Z1 adhesive).

**Figure 6:**
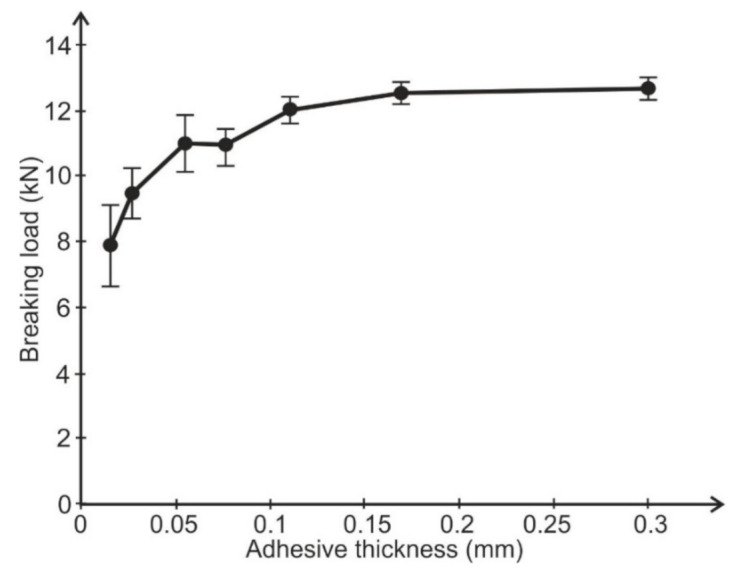
Dependence of lap joint strength on adhesive thickness (Araldite AW136H adhesive).

A verifying test was performed. A single simple lap joint with a 25 mm overlap, 25 mm width, and 2-mm-thick adherends made of aluminum alloy 2024T4 (Henan Mingtai aluminum industry Co. LTD, Zhengzhou, Henan, China) was tested to determine the influence of adhesive thickness upon joint strength. Six samples were used for each setting. The bonded surface of the specimens was prepared for bonding by pickling. Two thin wires were placed in uncured adhesive layers to obtain the definite adhesive thickness. The specimens were bonded with the same adhesives as the butt joints. The adhesive thickness was estimated by micrometer measuring of each specimen. The spread of average adhesive thickness was not greater then ±0.01 mm for each batch. The results of the strength tests are shown in Figure 5 and Figure 6.

The results of the experimental tests were different from the analytical calculations—the optimal adhesive thickness for joints bonded with Epidian 57 were smaller than the calculated ones, and the joints bonded with Araldite AV 136H did not show the optimal adhesive thickness. The cause of this inaccuracy may be the simplifications made, especially the assumptions with respect to the elastic strains of adhesive and adherends.

## 4. Numerical Calculations

FE method calculations were conducted to estimate the adhesive failure stresses in the adhesive layer of the experimentally tested single lap joints. As stated in the introduction, the authors decided to apply the continuum mechanics approach, and the adhesive joint was modeled with eight-node quad elements. The failure stress was calculated for joints bonded with Epidian 57 and AW 136H adhesives for the actual adhesive thickness and breaking loads on the basis of the experimental test. The MSC Nastran 2018 (MSC Software Corporation, Irvine, CA, USA) for Windows program was used for numerical calculation. In the numerical calculation, constant stress along the width of the joint was assumed. Therefore, the 2D plane strain models were used throughout this study. The actual thickness of specimen sheets, overlap lengths, thickness of the adhesive layer and the spacing of the testing machine handles were used in the numerical calculations. The adhesive layers were modeled by one layer of finite elements (eight-node quad elements) [30]. The possibility of such modeling is a result of the numerical investigations of lap joints subjected to shearing. The performed calculations led to the following conclusions:

- the edges of the adhesive layer are a so-called singular point in which numerically calculated stresses approach infinity when the element net is thickened,
- small (equal in dimension to the thickness of the adhesive layer) “flashes” presenting at the edges of the adhesive layers of the lap joint cause considerable reduction of the stress concentrations in adhesive layers, as well as the uniformity of stresses along the thickness of the adhesive layer,
- the uniformity of stresses along the thickness of the adhesive layer makes it possible to model the adhesive layer with a single element layer.

**Figure 7:**
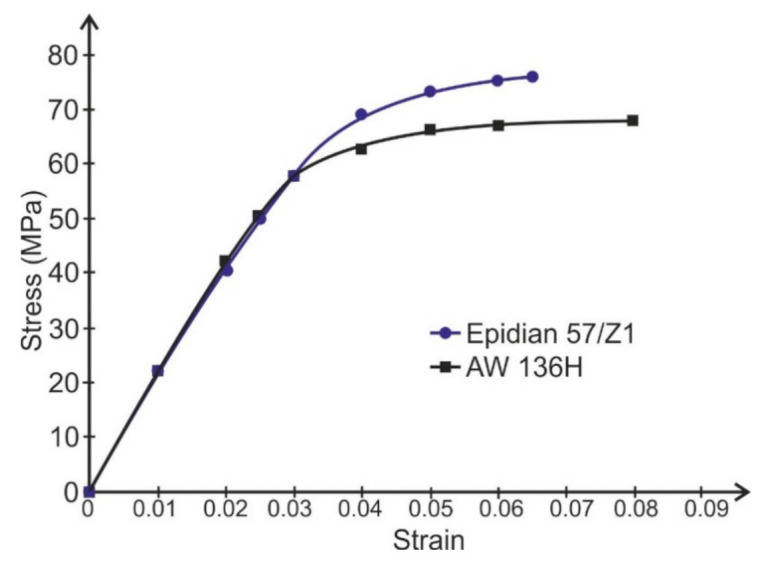
The stress–strain curves for Epidian 57/Z1 and AW 136H adhesive (compression curves without the considering of specimen cross-section area change).

**Figure 8:**
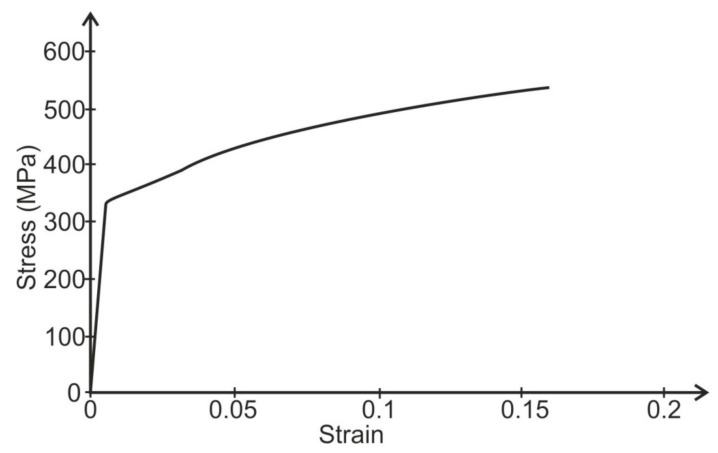
The stress–strain curve for 2024T4 alloy (tensile curve with the considering of specimen cross-section area change).

**Figure 9:**
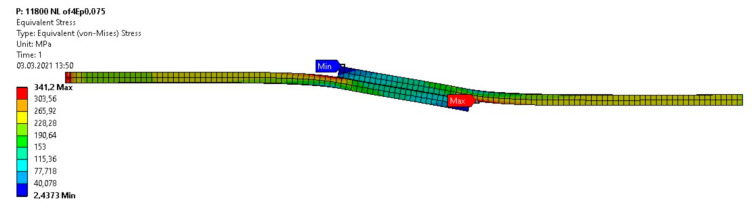
Deformation of a single lap specimen under shear load with a force of 11,800 N (scale 3.2:1, plastic deformation of jointed elements) and Mises stress distribution.

The length of the adhesive layer elements was equal to 0.5 mm. The adherends were modeled using four layers of elements. The tested models of the specimens were loaded with average forces according to the experimental tests in the respective groups. The calculations were performed for the non-linear characteristics of the adhesive and the adherends. The non-linear properties of the adhesive and the adherends were described for calculations using discrete functions made on the basis of their stress–strain curves. The stress–strain curves of adhesive layers (Figure 7) were adopted on the basis of the compression curves determined using the cast cylindrical specimens (diameter Φ = 12.5 mm, height l = 25 mm). The stress–strain curve of alloy 2024T4 based on the set of tensile curves is presented in Figure 8. The Poisson’s ratio of the alloy 2024T4 was assumed to be ν = 0.3 and the Poisson’s ratio of the adhesive was ν = 0.35. The model of the single lap specimen under load is illustrated on Figure 9.

**Figure 10:**
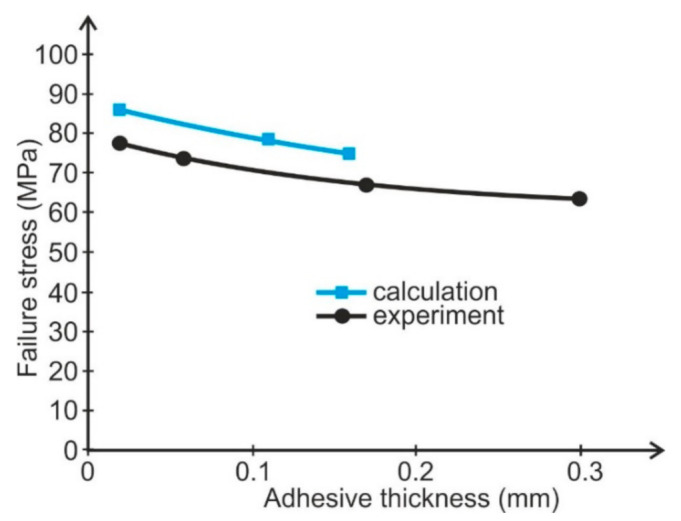
Comparison of Epidian 57/Z1 adhesive failure stresses determined experimentally using butt specimens with FEM calculated failure stresses for lap joints.

**Figure 11:**
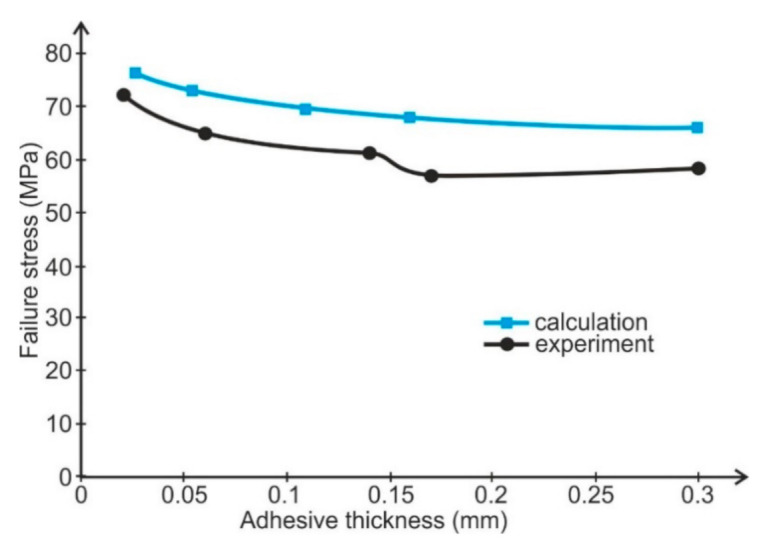
Comparison of AW 136H adhesive failure stresses determined experimentally using butt specimens with FEM calculated failure stresses for lap joints.

From the results of the conducted calculations, in all calculated cases, the yield point of adherends (Re ≈ 330 MPa) exceeded what caused plastic deformation. Calculation on the basis of the failure stress in the maximal principal stresses hypothesis showed results about 10% higher than the results from the tests of the tensile butt specimens (Figure 10 and Figure 11).

The cause of the difference in failure stress between the calculated and experimentally determined results may result from the greater area of the adhesive layer loaded with maximal stresses in the tensile butt joint than in the lap joint subjected to shearing. In the tensile butt joint, the entire adhesive is loaded by failure stress; in lap joint, only the edge of the adhesive layer is loaded. This is in accordance with defect theory relating to adhesive layers.

## 5. Conclusions

- The value of adhesive failure stress is dependent upon the adhesive thickness. The failure stress decreases with increasing adhesive layer thickness, at first in a quasi-linear manner, and then less intensively. This fact may be the result of the greater number of defects in the thicker adhesive layer.
- The dependence of adhesive failure stress upon adhesive thickness causes the strength of most adhesive joints (shear loaded and peel loaded) to be dependent on the thickness of the adhesive layer in different ways to those in the results shown by analytical formulas. Lap joints subjected to shearing and joints subjected to peeling may have the optimal adhesive thickness.
- The same adhesive joints, such as butt joints subjected to tensioning and joints subjected to cleaving, have the highest strength for very thin adhesive layers. For such joints, the facetious saying is “the adhesive bond is superlative if there is no adhesive at all”.
- The more accurate analysis of adhesive thickness influence on adhesively bonded joint strength demands the taking into account of the non-linear behavior of adhesive and the plastic strain ability of the adherends. This is possible using FE methods. The numerical calculations verify that the failure stress of thin adhesive layers is greater than in thicker ones.

## Figures and Tables

**Table 1:** Simplified formulas of adhesive joint coefficient for different joint loading valid for elastic adhesive (calculated on the basis of papers [15,16,17,18,19,20]).

No.	Model of Joint	Strength of Joint	Joint Coefficient	References
1	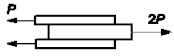	$P=\tau_{n}A$ *Ψ*_1_	$\psi_{1}=\sqrt{\frac{2E\delta \delta_{k}}{l^{2}G_{k}}}$	[21]
2	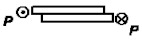	$P=\tau_{n}A$ *Ψ*_2_	$\psi_{2}=\sqrt{\frac{2G\delta \delta_{k}}{l^{2}G_{k}}}$	[22]
3	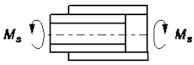	$M_{s}=\tau_{n}A$ *Ψ_3_*	$\psi_{3}=\frac{d}{l}\sqrt{\frac{G\delta \delta_{k}}{G_{k}}}$	[23]
4	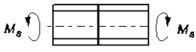	$M_{s}=\tau_{n}A$ *Ψ_4_*	$\psi_{4}=\frac{d}{2}$	*
5	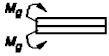	$M_{g}=\sigma_{n}A$ *Ψ*_5_	$\psi_{5}=\sqrt{\frac{E\delta^{3}\delta_{k}}{24l^{2}E_{k}}}$	* [24]
6	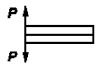	$P=\sigma_{n}A$ *Ψ*_6_	$\psi_{6}=\sqrt[{4}]{\frac{E\delta^{3}\delta_{k}^{}}{96l^{4}E_{k}}}$	* [26]
7	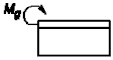	$M_{g}=\sigma_{n}A$ *Ψ*_7_	$\psi_{7}=\sqrt{\frac{E\delta^{3}\delta_{k}}{12l^{2}E_{k}}}$	[24,25]
8	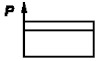	$P=\sigma_{n}A$ *Ψ*_8_	$\psi_{8}=\sqrt[{4}]{\frac{E\delta^{3}\delta_{k}^{}}{48l^{4}E_{k}}}$	[26]
9	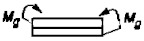	$M_{g}=\sigma_{n}A$ *Ψ*_9_	$\psi_{9}=\sqrt{\frac{E\delta^{3}\delta_{k}}{6l^{2}E_{k}}}$	* [24]
10	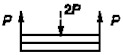	$P=\sigma_{n}A$ *Ψ*_10_	$\psi_{10}=\sqrt[{4}]{\frac{E\delta^{3}\delta_{k}^{}}{6l^{4}E_{k}}}$	* [26]
11	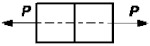	$P=\sigma_{n}A$ *Ψ*_11_	$\psi_{11}=1$	*
12	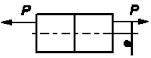	$P=\sigma_{n}A$ *Ψ*_12_	$\psi_{12}=\frac{d}{d+8e}$	*
13	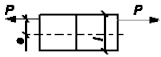	$P=\sigma_{n}A$ *Ψ*_13_	$\psi_{13}=\frac{l}{l+6e}$	*
14	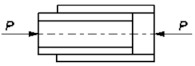	$P=\tau_{n}A$ *Ψ*_14_	$\psi_{14}=\sqrt{\frac{E\delta \delta_{k}}{2l^{2}G_{k}}}$	* [21]

*—own study, * [A]—own study based on publication [A].

## Data Availability

Data are contained within the article.
